# Insights into Metabolic Reprogramming in Tumor Evolution and Therapy

**DOI:** 10.3390/cancers16203513

**Published:** 2024-10-17

**Authors:** Ching-Feng Chiu, Jonathan Jaime G. Guerrero, Ric Ryan H. Regalado, Jiayan Zhou, Kin Israel Notarte, Yu-Wei Lu, Paolo C. Encarnacion, Cidne Danielle D. Carles, Edrian M. Octavo, Dan Christopher I. Limbaroc, Charupong Saengboonmee, Shih-Yi Huang

**Affiliations:** 1 Graduate Institute of Metabolism and Obesity Sciences, Taipei Medical University, Taipei 110301, Taiwan; jgguerrero1@up.edu.ph (J.J.G.G.); yuway120@gmail.com (Y.-W.L.); pcencarnacion1@up.edu.ph (P.C.E.); 2 Taipei Medical University Research Center of Cancer Translational Medicine, Taipei Medical University, Taipei 110301, Taiwan; 3 College of Medicine, University of the Philippines Manila, Manila 1000, Philippines; cdcarles@up.edu.ph (C.D.D.C.); emoctavo@up.edu.ph (E.M.O.); dilimbaroc1@up.edu.ph (D.C.I.L.); 4 College of Public Health, University of the Philippines Manila, Manila 1000, Philippines; 5 National Institute of Molecular Biology and Biotechnology, College of Science, University of the Philippines Diliman, Quezon City 1101, Philippines; rhregalado@up.edu.ph; 6 Department of Medicine, Stanford University School of Medicine, Stanford, CA 94305, USA; jyzhou@stanford.edu; 7 Department of Pathology, Johns Hopkins University School of Medicine, Baltimore, MD 21287, USA; kinotarte@gmail.com; 8 Department of Industrial Engineering and Management, Yuan Ze University, 135 Yuan-Tung Road, Chung-Li 32003, Taiwan; 9 Department of Biochemistry, Faculty of Medicine, Khon Kaen University, Khon Kaen 40002, Thailand; charusa@kku.ac.th; 10 School of Nutrition and Health Sciences, Taipei Medical University, Taipei 110301, Taiwan

**Keywords:** cancer, metabolic reprogramming, Warburg effect

## Abstract

**Background:** Cancer remains a global health challenge, characterized not just by uncontrolled cell proliferation but also by the complex metabolic reprogramming that underlies its development and progression. **Objectives:** This review delves into the intricate relationship between cancer and its metabolic alterations, drawing an innovative comparison with the cosmological concepts of dark matter and dark energy to highlight the pivotal yet often overlooked role of metabolic reprogramming in tumor evolution. **Methods:** It scrutinizes the Warburg effect and other metabolic adaptations, such as shifts in lipid synthesis, amino acid turnover, and mitochondrial function, driven by mutations in key regulatory genes. **Results:** This review emphasizes the significance of targeting these metabolic pathways for therapeutic intervention, outlining the potential to disrupt cancer’s energy supply and signaling mechanisms. It calls for an interdisciplinary research approach to fully understand and exploit the intricacies of cancer metabolism, pointing toward metabolic reprogramming as a promising frontier for developing more effective cancer treatments. **Conclusion:** By equating cancer’s metabolic complexity with the enigmatic nature of dark matter and energy, this review underscores the critical need for innovative strategies in oncology, highlighting the importance of unveiling and targeting the “dark energy” within cancer cells to revolutionize future therapy and research.

## Simple Summary

Cancer is a global health problem caused by uncontrolled cell growth and changes in how cells get and use energy. This review compares cancer’s hidden metabolic changes to dark matter and dark energy in the universe, which are mysterious and often ignored. It looks at how cancer cells alter their energy use, such as through the Warburg effect and changes in fat and protein production, driven by genetic mutations. These changes help cancer grow and survive. The review suggests that targeting these metabolic pathways could be a new way to treat cancer. It calls for more research to better understand these changes and develop new therapies by focusing on the "dark energy" that fuels cancer cells.

## 1. Introduction

Cancer represents a challenge on a global scale, exerting profound morbidity and mortality rates that strain healthcare financing and strategic planning worldwide [1]. The burden of cancer disproportionately impacts individuals from lower socioeconomic backgrounds and those experiencing adverse social determinants, underscoring the disease’s intricate interplay with broader societal inequalities [2,3,4]. The variability in cancer prognosis is attributed to the inherent complexity of the disease, which is further complicated by limited access to treatment and a lack of comprehensive research aimed at understanding its progression.

Cancer’s complexity is rooted in its nature as a multifaceted disease characterized by uncontrolled cell division and growth. This process is driven by a myriad of cellular and molecular mechanisms designed to evade the body’s homeostatic controls and exploit this aberrant behavior for the survival of cancer cells. Such complexity is further amplified by the disease’s heterogeneity, encompassing a vast array of types and subtypes, each defined by unique characteristics and behaviors. These often arise from the combination of genetic susceptibility and environmental exposures that cause mutations in key regulatory genes [5,6]. These alterations disrupt the balance between cell proliferation and apoptosis, enabling cancer cells to evade normal regulatory checks. Consequently, cancer cells acquire capabilities for sustained proliferative signaling, apoptosis resistance, and angiogenesis induction, which are critical for tumor progression.

Employing a metaphorical lens, the progression of cancer can be likened to the elusive concepts of “dark matter” or “dark energy” in cosmology. This analogy serves to illuminate the critical yet often obscured role of metabolic reprogramming in the survival and proliferation of cancer cells, akin to the foundational yet mysterious forces that govern the universe’s structure and expansion. The metabolic pathways of cancer, shrouded within complex biochemical networks, pose significant challenges for research, necessitating a strategic approach that transcends direct cellular targeting to include a nuanced understanding and manipulation of these metabolic processes.

Through an interdisciplinary lens that merges insights from biology, chemistry, physics, and computational sciences, we aim to highlight the intricate metabolic networks governing cancer cells. By drawing parallels with the study of dark matter and energy, we underscore the pivotal role of metabolic understanding in oncology, framing it as a frontier ripe with challenges yet abundant with potential for groundbreaking therapeutic advancements.

## 2. Metabolic Reprogramming in Cancer Cells

A hallmark feature of cancer is its metabolic reprogramming, which diverges significantly from the metabolic pathways observed in normal cells [7]. This reprogramming, exemplified by the Warburg effect, facilitates a preference for glycolysis over oxidative phosphorylation for adenosine triphosphate (ATP) production, despite the inefficiency of this process [8]. This metabolic shift is crucial for supporting the rapid energy and biomass production necessary for cancerous growth. Furthermore, cancer cells exhibit modifications in several other metabolic pathways, including oxidative phosphorylation, generation of reactive oxygen species, de novo lipid synthesis, fatty acid β-oxidation, glutaminolysis, and mitochondrial metabolism [9,10,11], highlighting the extensive metabolic flexibility that cancer cells employ to thrive.

**Figure 1:**
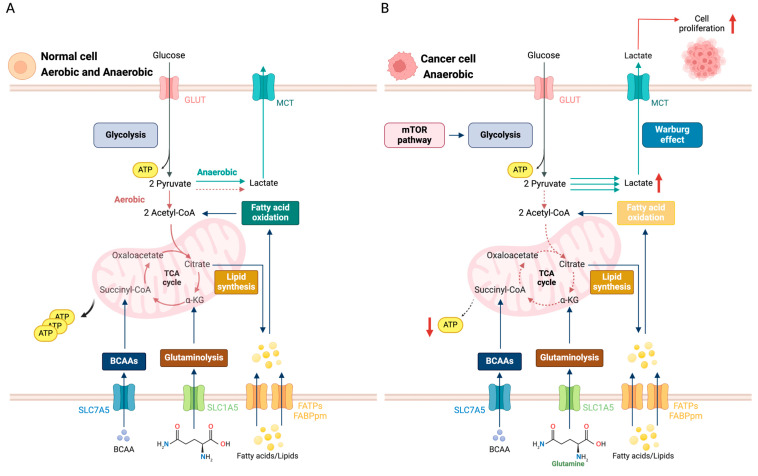
A comparison of the major metabolic pathways in (**A**) normal and (**B**) cancer cells. In normal cells (**A**), glucose enters via GLUT transporters, fueling glycolysis and predominantly generating ATP through oxidative phosphorylation (OXPHOS) in the mitochondria. Fatty acid oxidation and glutaminolysis also contribute to ATP production and lipid synthesis. Pathways with relatively low activity, such as lactate production, are indicated by dashed lines. In contrast, cancer cells (**B**) demonstrate increased glucose uptake via upregulated GLUT transporters, resulting in enhanced glycolysis and the Warburg effect, where pyruvate is converted to lactate even in the presence of oxygen. This metabolic reprogramming supports rapid ATP production and proliferation. Despite the dominance of the Warburg effect, minimal TCA cycle activity and OXPHOS are retained, as indicated by the dashed lines. Abbreviations: glucose transporter type 1 (GLUT1); Monocarboxylate Transporter (MCT); adenosine triphosphate (ATP); mammalian target of rapamycin (mTOR); α-Ketoglutarate (α-KG); branched-chain amino acids (BCAAs); Solute Carrier Family 7 Member 5 (SLC7A5); Solute Carrier Family 1 Member 5 (SLC1A5); Fatty Acid Transport Protein (FATP); Plasma Membrane Fatty Acid-Binding Protein (FABPpm).

Metabolism constitutes a balanced interplay between the assimilation and breakdown of molecular components orchestrated within the cytoplasm, mitochondria, and endoplasmic reticulum—key sites for a myriad of metabolic pathways. This equilibrium is essential for cell proliferation, necessitating upregulated metabolic activity to support the synthesis of proteins, nucleic acids, and lipids driven by growth factors [12]. A pivotal aspect of this regulatory mechanism is the enhanced uptake and utilization of glucose, culminating in glycolysis (Figure 1A) [13]. Under aerobic conditions, cells typically direct glucose-derived pyruvate through oxidative phosphorylation in the mitochondria for the efficient production of energy [14]. In contrast, under anaerobic conditions, pyruvate is used for lactate production [15].

Cancer cells, however, exhibit a marked deviation from this metabolic blueprint, favoring glycolysis even under aerobic conditions—a phenomenon less efficient in ATP production known as the Warburg effect (Figure 1B) [16]. This metabolic idiosyncrasy is not merely a peculiarity but a strategic adaptation, enabling rapid energy production and the synthesis of biomolecules critical for unrestrained growth. The molecular underpinnings of the Warburg effect reveal an overexpression of glucose transporters facilitated by glucose transporter type 1 (GLUT1) and glucose transporter type 3 (GLUT3), enabling glucose internalization, alongside upregulations in key glycolytic enzymes, indicative of systemic reprogramming toward aerobic glycolysis, as observed in hepatocellular carcinoma [17,18].

The Warburg effect is a hallmark of cancer metabolism, characterized by the conversion of glucose to lactate, albeit in the presence of oxygen-rich environment/conditions and functional mitochondria [19]. In the original work of Otto Warburg in 1924, he observed that the tumor tissue has decreased cellular respiration and produces a high amount of lactate [20]. Eventually, in 1956, Warburg posited that defects in the mitochondria were responsible for cancer and neoplasia [21]. Scientists have investigated the Warburg effect for many years as an effect of this debate. However, this idea has been disregarded in recent studies.

Over the years, different ideas have been published that add to the facets of the Warburg effect as a cancer metabolic phenomenon. A paper published by Luengo et al. [22] explained a different take of what was truly happening during the event. They argued that aerobic glycolysis happens when there is more demand for NAD+ compared to ATP, and cells shift toward fermentation instead of oxidative phosphorylation [22]. Another viewpoint in this debate posits that the citric acid cycle, rather than glycolysis, is the primary driver of cancer cell proliferation [23]. Additionally, some hypotheses focus on the role of the immune system in cancer development. Tsai and colleagues proposed that T cell-mediated immunosurveillance increases glucose uptake to produce lactate while suppressing oxidative phosphorylation in tumor cells [24]. Meanwhile, Otto Warburg’s assertion that mitochondrial dysfunction plays a critical role in cancer has been supported by findings, but is more than a simple adaptation [25]. Mutations in Krebs cycle enzymes, such as succinate dehydrogenase (SDH), fumarate hydratase (FH), and isocitrate dehydrogenase (IDH), contribute to cancer progression [26]. Despite a century of research, the molecular mechanisms underlying the Warburg effect remain an enigma.

## 3. Metabolic Pathways and Genetic Dysfunctions in Cancer

**Figure 2:**
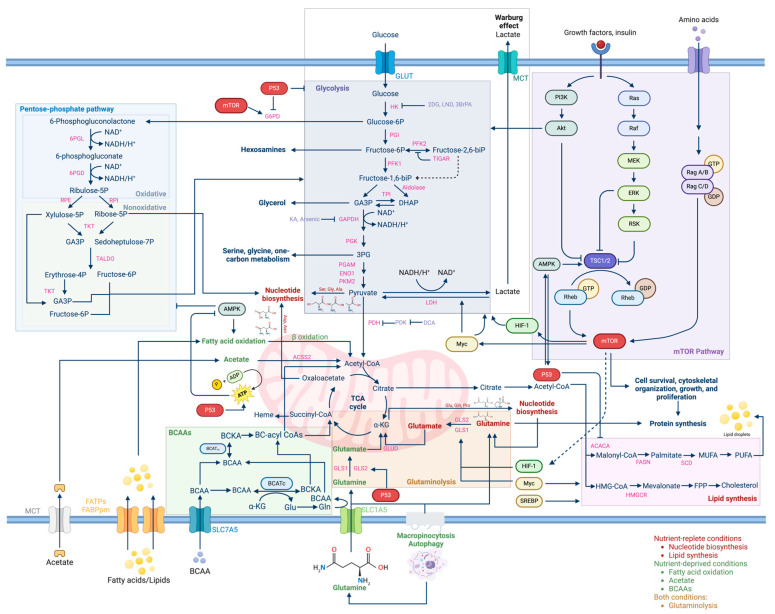
An overview of the major metabolic pathways at work within cancer cells. Cell survival, growth, and proliferation require glucose to generate ATP, lipids, and amino acids through glycolysis, alongside other downstream reactions and pathways, including the pentose phosphate pathway, glutaminolysis, lipid synthesis, and branched-chain amino acid (BCAA) metabolism. The Warburg effect, characterized by increased glucose uptake and lactate production despite adequate oxygen, highlights metabolic reprogramming, supporting rapid tumor growth and survival even under oxidative conditions. The mTOR signaling pathway regulates cell growth, proliferation, survival, and cytoskeletal organization in response to insulin, growth factors, and other metabolic and cellular cues. Additionally, p53 plays an important role in promoting ATP production, facilitating citric acid cycle (also referred to as the TCA cycle or Krebs cycle) and glutamate synthesis, while regulating glycolysis and lipid synthesis. Dysregulation of mTOR signaling and p53 has been implicated in numerous diseases, including cancer and metabolic disorders. Moreover, the metabolic processes of cancer cells operate in distinct ways depending on the availability of nutrients. In situations where nutrients are abundant (nutrient-replete conditions), there is a focus on nucleotide production, lipid generation, and the utilization of glutamine. Conversely, under nutrient-deprived conditions, cancer cells favor fatty acid oxidation, acetate breakdown, the utilization of BCAAs, and glutaminolysis related to macropinocytosis and autophagy. Understanding the metabolic adaptations of cancer cells to diverse nutrient environmental conditions is vital for developing targeted therapies to combat disease progression. Abbreviations: Pentose phosphate pathway. glucose-6-phosphate dehydrogenase (G6PD); Ribulose 5-phosphate (Ribulose-5P); Xylulose 5-phosphate (Xylulose-5P); Ribose 5-phosphate (Ribose-5P); Glyceraldehyde 3-phosphate (G3P); Sedoheptulose 7-phosphate (sedoheptulose-7P); Transaldolase (TALDO); Erythrose 4-phosphate (Erythrose-4P); Fructose 6-phosphate (Fructose 6-p). Glycolysis. glucose-6-phosphate dehydrogenase (G6PD); Fructose 6-phosphate (Fructose 6-p); Fructose 1,6-biphosphate (Fructose 1,6-biP); Fructose 2,6-biphosphate (Fructose 2,6-biP); Glyceraldehyde 3-phosphate (GA3P); Dihydroxyacetone phosphate (DHAP); Glyceraldehyde-3-phosphate dehydrogenase (GAPDH); Phosphoglycerate mutase (PGAM); Pyruvate kinase M2 (PKM2); Lactate dehydrogenase (LDH). mTOR pathway. Phosphatidylinositol-3 kinase (PI3K); Protein kinase B (AKT); Rat sarcoma (Ras); Rapidly Accelerated Fibrosarcoma (Raf); Mitogen-Activated Protein Kinase (MEK); Extracellular Signal-Regulated Kinase (ERK); p90 Ribosomal S6 Kinase (RSK); Tuberous Sclerosis Complex1/2 (TSC1/2); Ras Homolog Enriched in Brain (Rheb); Guanosine Triphosphate (GTP); Ras-related GTP binding A/B (Rag A/B); Ras-related GTP binding C/D (Rag C/D); Guanosine Diphosphate (GDP); mammalian target of rapamycin (mTOR); Hypoxia-Inducible Factor 1 (HIF-1). branched-chain amino acid (BCAA). α-ketoglutarate (α-KG); glutamine (Gln); glutamate (Glu); Branched-chain Aminotransferases (BCAT); 3-hydroxy-3-methyl-glutaryl-CoA (HMG-CoA); α-ketoisocaproate (KIC); branched-chain amino acid Aminotransferase (BCAT). Glutaminolysis. glutaminase (GLS); Glutamate Dehydrogenase (GLUD); Sodium-Dependent Neutral Amino Acid Transporter (SLC1A5). Lipid Synthesis. 3-hydroxyl3-methyl-glutaryl-coenzyme A reductase (HMG-CoA); Acetyl-CoA Carboxylase (ACACA); fatty acid synthase (FASN); 3-hydroxy-3-methylglutaryl-CoA Reductase (HMGCR); Farnesyl Pyrophosphate (FPP); Stearoyl-Coa Desaturase (SCD); Monounsaturated Fatty Acid (MUFA); Polyunsaturated Fatty Acid (PUFA).

Beyond glycolysis, cancer metabolism encompasses a broad spectrum of altered pathways (Figure 2). The tricarboxylic acid cycle (TCA), while still operational for oxidative phosphorylation, exhibits an increased demand for the intermediates essential for rapid biosynthesis and energy production, a demand often met through mutations in enzymes like SDH, FH, and IDH, leading to TCA dysregulation [27,28,29]. Glutaminolysis also emerges as a critical pathway, with cancer cells demonstrating a heightened reliance on glutamine, facilitated by transporters such as the Alanine, Serine, Cysteine transporter 2 (ASCT2) and catalyzed by the upregulation of glutaminases—a type of reprogramming observed in various cancers including prostate cancer [30,31,32].

Conversely, fatty acid β-oxidation acts as a bulwark against apoptosis, with the overexpression of enzymes like long-chain-fatty-acid-CoA ligase 4 (ACSL4) and alpha-methylacyl-CoA racemase (AMACR) enhancing mitochondrial integrity and cellular survival [33,34,35]. These processes are controlled genetically, and some of the key genes are listed in Table 1.

Despite advancements in our understanding, the metabolic landscape of cancer remains profoundly elusive, mirroring the mysterious nature of dark matter and dark energy in the cosmos. This elusiveness stems from a constellation of challenges that beset researchers endeavoring to decipher the complex pathophysiology underpinning cancer metabolism. The intricate metabolic processes operating within tumors are often as concealed as the cosmological phenomena, with only indirect evidence—such as the presence or absence of specific metabolites and enzymes—hinting at the underlying dysfunctions.

Cancer’s inherent heterogeneity further complicates this landscape. Each tumor presents a unique metabolic profile, influenced by its genetic makeup, microenvironment, and the specific mutations it harbors. This diversity means that while general patterns of metabolic reprogramming can be identified, as explored in this review, the reality is a mosaic of variations. Certain cancer cells, for instance, may preferentially utilize glucose or glutamine, reflecting a deviation from typical metabolic pathways [36]. Others, including gliomas, lung cancers, and leukemias, retain a reliance on oxidative phosphorylation to fuel rapid proliferation [37,38]. The targeting of reactive oxygen species (ROS) in chemotherapy highlights the unpredictable nature of cancer metabolism, with treatments sometimes exacerbating malignancy due to the varied redox responses across different tumors [39].

**Table 1:** Genetic control and dysfunctions in cancer metabolic reprogramming.

Metabolism	Oncogenic Protein	Metabolic Enzyme Targets	Mechanisms and Phenotype
Glycolysis, glutaminolysis, and amino acid synthesis	Myc	GLUT, HK2, and PFK [40,41]; LDH and MCT1 [42,43]; SLC1A5 and SLC38A5 [44]; GLS [45]; GLUD and transaminase [46,47]; G6PD and TKT [48]	Gain-of-function mutation enhances cell cycle progression and metabolism in cancer by upregulating the expression of glucose transporters and the majority of glycolytic enzymes, promoting glycolysis and glutaminolysis.
Glycolysis, tricarboxylic acid cycle, and fatty acid oxidation	p53	GLUT1/4 [49]; TIGAR [50]; LDH and PDH [51]; CPT1 and LPIN1 [52,53]	A loss of p53 alters the metabolism in cancer cells by downregulating several enzymes and transporters inhibiting mitochondrial respiration, glycolysis, and apoptosis.
Fatty acid synthesis and glycolysis	PTEN	PI3K/AKT [8]	Mutations or a loss of PTEN result in negative regulation of the PI3K/AKT signaling pathway and in turn intracellular metabolic reprogramming, promoting the growth and proliferation of cancer cells.
Glycolysis, glutaminolysis, and amino acid synthesis	Ras	PI3K/AKT/mTOR [8]; RAF/MEK/ERK [8]; Myc [8];	Oncogenic mutations lead to the upregulation of enzymes, resulting in tumor metabolic reprogramming and promotion of cell proliferation and survival.
Glycolysis and fatty acid synthesis	PIK3CA	PI3K/AKT [8]	Mutations lead to the activation of the PI3K/AKT pathway and enhance intracellular signal transduction, which leads to subsequent metabolic reprogramming of cancer cells.
Glycolysis and glutaminolysis	EGFR	PI3K/AKT; RAF/MEK/ERK [54]	EGFR signaling pathways activate lipogenesis through PI3K/AKT and MAPK pathways, leading to increased de novo lipid synthesis and alterations in lipid metabolism that support cancer cell growth and proliferation.
Glycolysis	PDK1	PDHC [55]	Activation promotes a shift from oxidative phosphorylation to glycolysis by inhibiting the pyruvate dehydrogenase complex, thereby redirecting cellular metabolism to support tumorigenesis and metastasis.
Glycolysis, de novo lipogenesis, and protein synthesis	NF1	Neurofibromin [56]	Loss-of-function mutations alter neurofibromin expression, increase RAS and PI3K/AKT pathway signaling, constraining oxidative ATP production, restrict energetic flexibility, and increase glutamine influx into TCA intermediates, expanding lipid pools (especially triglycerides) and altering the synergy between metabolic inhibitors and traditional targeted inhibitors.
Glycolysis, tricarboxylic acid cycle, and fatty acid synthesis	HIF-1α	HK2, PDK1, LDHA [57,58]	In response to hypoxia, HIF-1α upregulates the activation of genes involved in glycolysis and metabolism, cell proliferation, angiogenesis, invasion, and metastasis.
Glycolysis, protein synthesis, and lipid metabolism	TSC2	Rheb [59]	Loss-of-function mutations lead to abnormal activation of the mTOR pathway through increased Rheb activity. This results in altered protein synthesis, lipid metabolism, and glucose metabolism.
Glycolysis and fatty acid oxidation	SIRT1	β-catenin [60]	When upregulated in response to glucose deficiency and oxidative stress, SIRT1 deacetylates β-catenin, causing its translocation from the nucleus to the cytoplasm, attenuates glycolysis, and positively correlates with fatty acid oxidation. This promotes the shift in glycolipid metabolism, facilitating tumor development in colorectal carcinoma.
Glycolysis, amino acid metabolism, lipid metabolism, and bile acid metabolism	YAP/TAZ	GLUT3 [61]; HK2 [62]; PFKFB3 [63]; SLC1A5 and SLC7A5 [64,65]; GOT1 and PSAT1 [66,67]	Overactivation promotes glycolysis by increasing GLUT3, HK2, and PFKFB3 expression, enhancing glutamine metabolism by upregulating transporters and enzymes. It modulates lipid and bile acid accumulation, aiding cancer metastasis.
Glycolysis and fatty acid oxidation	LKB1	AMPK 1/2, MARK 1/2/3/4, SIK 1/2/3, NUAK 1/2, and SNRK [68]	LKB1 deficiency leads to the dysregulation of cellular energy homeostasis and contributes to the metabolic reprogramming of cancer cells, which induces excess glycolysis, the primary energy supply for cancer cells, enhancing their cellular growth and proliferation.
Glycolysis and tricarboxylic acid cycle	FH	PDHA1 [69]	Mutations lead to metabolic reprogramming characterized by increased glycolytic flux, a shift to glutamine as the primary carbon source, the induction of pseudohypoxia, alterations in lipid biosynthesis, and enhanced arginine metabolism, collectively promoting a favorable environment for cancer progression.
Glycolysis	PGAM1	Wnt/β-catenin [70]; BCL-2, BAX, and caspase-3 [71]; ACTA2 [72,73]	Overexpression results in dysregulated glycolysis, leading to altered bioenergetics characterized by increased aerobic glycolysis (Warburg effect), thereby promoting cancer cell growth, proliferation, and invasion.
Tricarboxylic acid cycle	IDH1/2	TET2 [74]; JMJD2A [75]	Mutations lead to altered enzyme function, promoting the production of 2-hydroxyglutarate (2HG) which inhibits enzymes that cause differentiation in hematopoietic cells and histone methylation.

Abbreviations: Myelocytomatosis oncogene (Myc); glucose transporter type 2 (GLUT2); Hexokinase 2 (HK2); Phosphofructokinase (PFK); Lactate Dehydrogenase (LDH); Monocarboxylate Transporter 1 (MCT1); Solute Carrier Family 1 Member 5 (SLC1A5); Solute Carrier Family 7 Member 5 (SLC7A5); Solute Carrier Family 38 Member 5 (SLC38A5); glutaminase (GLS); Glutamate Dehydrogenase (GLUD); glucose-6-phosphate dehydrogenase (G6PD); Transketolase (TKT); TP53-Induced Glycolysis and Apoptosis Regulator (TIGAR); Pyruvate Dehydrogenase (PDH); Carnitine Palmitoyltransferase 1 (CPT1); Lipid Phosphatase and Proteins Phosphatase 1 (LPIN1); Phosphatase and Tensin Homolog (PTEN); Phosphoinositide 3-Kinase/Protein Kinase B (PI3K/AKT); Rat Sarcoma (Ras); mammalian target of rapamycin (mTOR); Rapidly Accelerated Fibrosarcoma/Mitogen-Activated Protein Kinase/Extracellular Signal-Regulated Kinase (RAF/MEK/ERK); Phosphoinositide 3-Kinase Catalytic Subunit Alpha (PIK3CA); Epidermal Growth Factor Receptor (EGFR); Pyruvate Dehydrogenase Kinase 1 (PDK1); Pyruvate Dehydrogenase Complex (PDHC); Neurofibromin 1 (NF1); Hypoxia-Inducible Factor 1 Alpha (HIF-1α); Lactate Dehydrogenase A (LDHA); Tuberous Sclerosis Complex 2 (TSC2); Ras Homolog Enriched in Brain (Rheb); Sirtuin 1 (SIRT1); Yes-Associated Protein/Transcriptional Coactivator with PDZ-Motif (YAP/TAZ); Phosphofructokinase Fructose-Bisphosphatase 3 (PFKFB3); Glutamate Oxidotransferase 1 (GOT1); Phosphoserine Aminotransferase 1 (PSAT1); Liver Kinase B1 (LKB1); AMP-Activated Protein Kinase 1/2 (AMPK 1/2); Microtubule Affinity-Regulating Kinase 1/2/3/4 (MARK 1/2/3/4); Salt-Inducible Kinase 1/2/3 (SIK 1/2/3); NUAK Family Kinase 1/2 (NUAK 1/2); Serine/Threonine/NORE1-Related Kinase (SNRK); Fumarate Hydratase (FH); Pyruvate Dehydrogenase Alpha 1 (PDHA1); Phosphoglycerate Mutase 1 (PGAM1); B-cell lymphoma 2 (BCL-2); Bcl-2-Associated X Protein (BAX); Alpha-Smooth Muscle Actin (ACTA2); Isocitrate Dehydrogenase 1/2 (IDH1/2); Tet Methylcytosine Dioxygenase 2 (TET2); Jumonji Domain-Containing 2A (JMJD2A).

Addressing this metabolic enigma requires innovative technological approaches. At the forefront are genetic technologies and structural biology, which offer insights into the genetic aberrations driving metabolic dysregulation in cancer cells [76]. Metabolomics, particularly when coupled with mass spectrometry, has emerged as a pivotal tool in mapping the cancer metabolome, facilitating both targeted and global analyses of cellular metabolites [77,78]. These methodologies, alongside isotope tracing techniques, are instrumental in tracing metabolic pathways, shedding light on how specific disruptions contribute to cancer progression [79,80].

## 4. The Tumor Microenvironment

As noted previously, each tumor presents a unique metabolic profile shaped by its genetic makeup, microenvironment, and specific mutations. This diversity creates a mosaic of variations, despite the identification of general patterns of metabolic reprogramming. A critical factor influencing these metabolic adaptations is the tumor microenvironment (TME), particularly under conditions of nutrient deprivation.

Central to this adaptability are key metabolic pathways, including fatty acid β-oxidation. Cancer cells have been shown to exploit lipid metabolism, enhancing lipid oxidation to thrive in hypoxic and nutrient-scarce conditions. This metabolic shift not only supports energy production but also alters the tumor microenvironment, promoting immunosuppression and cancer progression [81,82]. Oncogenes and mutated enzymes further contribute to these adaptations, enabling cancer cells to modify their microenvironment for continued growth and survival [83].

The pentose phosphate pathway (PPP) and fatty acid β-oxidation further illustrate cancer cells’ metabolic versatility. The PPP, vital for the synthesis of ribonucleotides and NADPH, induces an upregulation of glucose-6-phosphate dehydrogenase (G6PD) in cancerous tissues, a response to the oxidative stress endemic in tumor microenvironments [33,34,35,36,37].

Additionally, studies show that cancer proliferation often arises from competition between tumor cells and T cells for glucose in glucose-deficient environments, leading to increased glucose consumption by both cell types [25]. This metabolic tug of war underscores the adaptability of cancer cells in response to limited resources within the tumor microenvironment.

The TME is a complex and dynamic ecosystem that significantly influences cancer progression and therapeutic response. Within this milieu, cancer cells exhibit remarkable metabolic adaptability, allowing them to thrive under conditions of hypoxia and nutrient scarcity. The competition for limited resources, particularly glucose, between tumor cells and immune cells further complicates the metabolic landscape of the TME. This intricate interplay not only facilitates cancer cell survival and proliferation but also contributes to immunosuppression, thereby fostering a microenvironment conducive to tumor growth and metastasis. However, these changes are only part of the broader metabolic landscape of cancer. Another critical component in this reprogramming is the role of mitochondria, which, beyond their classical functions, have emerged as key players in cancer metabolism and will be the focus of the following section.

## 5. Mitochondria

Mitochondria play a multifaceted role in cancer evolution, influencing energy metabolism. They are vital in cellular signaling, apoptosis, and ROS production. While cancer cells primarily rely on aerobic glycolysis to support rapid proliferation, mitochondria remain crucial for biosynthesis, NAD+ regeneration, and redox balance [84,85]. Alterations in mitochondrial function can promote oncogenesis by generating excess ROS, which can cause DNA damage, activate oncogenes, and enhance genomic instability. This interplay between mitochondrial dysfunction and ROS production highlights mitochondria as key players in tumorigenesis [86].

The regulation of apoptosis by mitochondria is central to cancer development and resistance to therapy. In normal cells, mitochondrial outer membrane permeabilization (POMP) triggers the release of cytochrome c, leading to apoptosis. However, in cancer cells, this pathway is often suppressed through mutations in mitochondrial proteins or the overexpression of anti-apoptotic factors like B-cell lymphoma 2 (BCL-2) [87,88]. Such alterations allow cancer cells to evade cell death, contributing to their survival and resistance to conventional therapies, including chemotherapy and radiation [89]. Targeting mitochondrial pathways to restore apoptotic signaling is an emerging therapeutic strategy in cancer treatment, with promising compounds under investigation such as histone deacetylase inhibitors [90] and antisense oligonucleotides [91,92]. Some are shown in Table 2.

Another rapidly advancing area of research is mitochondrial dynamics. The processes of fission and fusion of mitochondria are closely linked to cancer progression and metastasis [98,99]. Mitochondrial fission is often upregulated in cancer cells, facilitating cell division, migration, and invasion [100]. Dysregulated fission, mediated by proteins such as dynamin-related protein 1 (DRP1), has been shown to promote metastasis in various cancers [98,99]. Targeting mitochondrial dynamics, along with mitochondrial DNA (mtDNA) mutations, represents a novel approach, as recent studies have demonstrated that disrupting mitochondrial fission can inhibit cancer cell proliferation and metastasis [100]. These findings indicate that mitochondria are not only essential for cancer cell metabolism but also for their survival, making them promising targets for cancer therapies.

## 6. Impact of Metabolic Reprogramming on Cancer Progression

Metabolic reprogramming plays a significant role in cancer progression, fueling the rapid proliferation and growth characteristics of malignancies. This reprogramming facilitates an enhanced reliance on glycolysis, providing a quick source of energy essential for tumor development. Glycolysis has been implicated in early tumorigenesis, driving epigenetic modifications, inhibiting cellular senescence, and enhancing DNA damage repair mechanisms, thereby supporting the survival and proliferation of cancer cells [101]. RAC-alpha serine/threonine-protein kinase (AKT), for instance, promotes glycolysis while exerting anti-apoptotic effects, further contributing to cancer cell survival [102]. Additionally, glycolytic metabolites such as lactate and pyruvate mediate interactions between cancer cells and their microenvironment, ultimately promoting tumor growth and metastasis [103]. Lactate, in particular, has been shown to induce polarization of tumor-associated macrophages toward an M2 phenotype, which supports tumor metastasis [104].

Amino acid metabolism also contributes to tumor metastasis and drug resistance. Hyperactivation of amino acid pathways is vital for biosynthesis in metastatic processes, with glutamine, serine, glycine, and proline playing key roles in maintaining metabolic intermediates essential for cancer development [105,106,107]. Proline catabolism, in particular, generates ROS that promote angiogenesis, signaling cascades, and epithelial-to-mesenchymal transition (EMT), which are hallmarks of aggressive cancer behavior [108].

Furthermore, the availability of amino acids is intricately linked to cancer cells’ epigenetic status and their capacity to develop drug-resistant phenotypes. For example, the increased uptake of glutamine alongside glucose has been associated with oral cancers’ resistance to cisplatin, while the production of putrescine via ornithine decarboxylase (ODC) contributes to erlotinib resistance [109,110]. Resistance to epidermal growth factor receptor (EGFR) tyrosine kinase inhibitors, often achieved through metabolic reprogramming of branched-chain amino acids, further illustrates the complex interplay between metabolism and drug resistance [111].

## 7. Interdisciplinary Approaches in Cancer Metabolism Research

The multifaceted nature of cancer demands an interdisciplinary approach, bridging biology, chemistry, physics, and computational sciences with the personal dimensions of the disease and its economic implications. Moreso, the economic impact and investments required to translate research to therapies are considerations worthy of discussion.

The variability in metabolic profiles across cancer types necessitates precision metabolomics, integrating metabolic data with transcriptomics, genomics, and proteomics to tailor treatments more effectively. This multi-omics strategy has shown the roles of specific proteins in cancers, such as chromobox protein homologs (CBX2 and CBX7) in breast cancer, and has facilitated the development of targeted therapies for conditions like clear cell renal carcinoma [112,113]. Furthermore, metabolomics has advanced our understanding of the metabolic distinctions between normal and cancerous tissues, aiding in the identification of novel biomarkers and therapeutic avenues [114,115,116].

Nutrigenomics and dietary interventions explore the interactions among nutrition, genetics, and metabolism, highlighting how diet can influence epigenetic modifications and affect disease outcomes. This area of research promises to enhance the precision of medical treatments through dietary adjustments [117,118,119]. Additionally, computational systems biology has played a crucial role in elucidating the dynamics of metabolic reprogramming, offering tools for simulation and analysis that deepen our understanding of cancer’s metabolic complexity [120,121].

## 8. Therapeutic Implications and Future Directions

Our expanding knowledge of cancer’s metabolic reprogramming has led to the identification of numerous potential therapeutic targets. Compounds designed to inhibit specific metabolic pathways, such as glutaminase inhibitors and mTOR inhibitors, are showing promise in clinical settings, addressing the metabolic vulnerabilities of cancer cells [122,123,124]. The use of rapamycin and its analogs in various cancers exemplifies the potential of targeting metabolic pathways to inhibit tumor growth. Additionally, metformin, traditionally used for diabetes management, is being explored for its anticancer properties, demonstrating the crossover potential of drugs across different therapeutic areas [125,126,127]. Some more therapeutic compounds at different stages of investigation are listed in Table 3.

## 9. Challenges and Limitations in Understanding and Targeting Cancer Metabolism

Recent insights into cancer biology have unveiled a complex and intricately organized structure governing tumor progression, challenging the traditional view of cancer as a disordered and chaotic entity. This organization suggests that similar to the cosmic influence of dark matter and dark energy, a hidden order underpins the metabolic reprogramming of cancer cells, offering novel perspectives for cancer medicine. Despite these advancements, the integration of cancer’s metabolic “dark energy” into clinical practice remains an elusive goal, reflecting the complexity of the disease and the limitations of current therapeutic approaches.

It is now evident that cancer’s metabolic network is not reliant on a singular pathway but involves complex interactions among multiple inter-related pathways [149,150]. This heterogeneity is compounded by genetic alterations in oncogenes and tumor suppressor genes, which drive the metabolic rewiring essential for tumorigenesis and malignant transformation [12,151]. Yet, the translation of this knowledge into effective therapies is hindered by the complex nature of cancer metabolism and the inherent limitations of existing treatment modalities.

Traditional cancer therapies, such as chemotherapy and radiotherapy, suffer from significant drawbacks, including systemic toxicity and a lack of selectivity, often damaging healthy tissues alongside cancer cells [152]. This issue is exacerbated by the cancer cells’ ability to activate self-renewal pathways and metabolic shifts that favor tumor growth, further complicating the development of targeted therapies. Although small-molecule inhibitors have shown promise, their clinical application is limited by issues such as nonspecific toxicity and poor solubility [153]. The redundancy and crosstalk among signaling pathways necessitate a multifaceted therapeutic approach, rather than targeting single pathways. For example, CD147 or the extracellular matrix metalloproteinase inducer (EMMPRIN) is one target candidate in hematological malignancies [154]. Single-cell metabolomics, focusing on metabolic rewiring at the cellular level, is also an approach worth researching, as this may have potential, as shown in hematologic malignancies [155].

A critical challenge in cancer treatment is tumor heterogeneity and the dynamic nature of cancer metabolism, which vary not only among patients but also within individual tumors. This metabolic flexibility allows cancer cells to adapt to environmental pressures and treatment interventions, making them moving targets for therapy. The variability in metabolic programming underscores the need for personalized treatment strategies that consider the unique metabolic landscape of each patient’s cancer.

To overcome these challenges, a comprehensive understanding of the molecular mechanisms underlying cancer’s resilience and adaptability is essential. As our knowledge of the cosmos expands, so too must our understanding of cancer’s complexity. Future therapeutic strategies must prioritize the development of more accurate models that reflect the tumor microenvironment’s intricacy and the pivotal role of metabolism in cancer progression. Embracing this complexity and adopting a more nuanced approach to cancer metabolism will be crucial for advancing the development of effective, clinically translatable cancer therapies.

## 10. Conclusions

By drawing parallels with cosmological concepts like dark matter and dark energy, this review presents the hidden dimensions of cancer cell metabolism and highlights the importance of understanding its complexities. Moving forward, interdisciplinary research efforts and innovative strategies are needed to fully exploit this frontier. Ultimately, unveiling and targeting the “dark energy” within cancer cells could revolutionize future therapy and research, offering hope for more effective and clinically sound treatments.

## Figures and Tables

**Table 2:** Compounds or drugs targeting mitochondrial pathways to restore apoptosis.

Compound or Drug	Mechanism	Target	Specific Cancer	Findings	Clinical Pipeline	Reference
Entinostat	Inhibits the function of histone deacetylases leading to more relaxed chromatin and gene expression	HDAC1/3	Osteosarcoma	Shown to upregulate the expression of Fas, leading to decreased pulmonary metastasis and improved outcomes	Preclinical	[93]
Venetoclax	Selective BCL-2 inhibitor that releases pro-apoptotic proteins	BCL-2	Acute myeloid leukemia	Overall response rate of 67% when combined with hypomethylating agents in elderly AML patients	FDA-approved, Phase III	[94]
MCL-1- specific inhibitor AZD5991	Binds directly to Mcl-1 and induces rapid apoptosis in cancer cells by activating the Bak-dependent mitochondrial apoptotic pathway	MCL-1	Myeloma and acute myeloid leukemia	Induces apoptosis in >80% of multiple MCL-1-dependent myeloma cell lines in vitro	Preclinical	[95]
Lenvatinib	Induces immunogenic cell death and activates TLR 3/4 ligands, enhancing immune response	TLR 3/4	Hepatocellular carcinoma	Triggers immunogenic cell death, enhancing anti-tumor immunity; increases T-cell activation and infiltration by 40% in HCC models	Preclinical	[96]
Obatoclax	Directly induces apoptosis through the activation of BAX/BAK following their release from the pro-survival BCL-2 members	Pan-BCL -2 family	Hematologic malignancies	Demonstrates tolerability and partial responses in patients with chronic lymphocytic leukemia and Hodkins lymphoma	Phase I clinical trial	[97]

Abbreviations: histone deacetylase 1/3 (HDAK1/3); B-cell lymphoma 2 (BCL-2); Myeloid Cell Leukemia 1 (MCL-1); acute myeloid leukemia (AML); Toll-like Receptors 3/4 (TLR3/4); hepatocellular carcinoma (HCC); Bcl-2-associated X protein/Bcl-2 antagonist/killer (BAX/BAK).

**Table 3:** Therapeutic compounds or drugs and targets within cancer metabolism.

Metabolism	Compound or Drug	Target	Mechanism	Findings	Clinical Pipeline	Reference
Glycolysis	3-Bromopyruvate	Hexokinase II	Irreversibly alkylates HK2, resulting in the disruption of glucose metabolism, leading to cancer cell death	3-BP (20 mg/kg) reduced tumor size by 75–80% in animals and induced apoptosis and necrosis in drug-treated tumor tissues	Animal Studies	[128]
TCA cycle	6-Methoxydihydroavicine (6-ME)	Oxaloacetic Acid Metabolism	Disrupts OAA metabolism, leading to ROS accumulation and resulting in disrupted mitochondrial homeostasis, ultimately driving apoptosis in ovarian cancer cells	6-ME significantly reduced tumor growth in a nude mouse model of ovarian cancer without causing physiologically harmful effects on the animal	Animal Studies	[129]
Glycolysis	AZD3965	MCT-1	Inhibition of MCT1-mediated lactic acid efflux during T-cell lymphocyte proliferation	The drug showed rapid oral absorption, nearly complete bioavailability, nonlinear pharmacokinetics, and potential involvement in enterohepatic circulation (EHC), with evidence of target-mediated drug disposition (TMDD)	Phase I	[130]
Glycolysis	Benserazide (Benz)	Hexokinase II	Competitive and noncompetitive binding to selectively inhibit HK2	In vivo, it suppresses tumor growth in mice without toxicity; when formulated as liposomal nanoparticles, Benz enhances tumor targeting and efficacy at lower doses	Animal Studies	[131]
Glutaminolysis	CB839 (Telaglenastat)	Glutamine oxidase	Block glutamine-to-glutamate conversion, reducing the number of immunosuppressive cells and reshaping the tumor microenvironment	The study established a recommended phase II dose (RP2D) for telaglenastat, demonstrating safety, strong GLS inhibition, and early anticancer activity, prompting further investigation	Phase I	[132]
Glycolysis	Curcumin (Cur) + Thymoquinone (TQ)	Caspase-3 and PI3K/AKT	Induces apoptosis and cell cycle arrest, and decreases proliferation, colony formation, and migration of MCF7 and MDA-MB-231 cells	Cur and TQ significantly inhibited cancer cell growth and migration, increased apoptosis (73.96% for Cur, 75.76% for Cur + TQ), and reduced S-phase values compared to controls	In vitro	[133]
Glycolysis	Demethylzeylasteral (DML)	Lactate	Dose-dependent decrease in intracellular lactate levels Regulation of histone acetylation via H3K9la and H3K56la modification sites	DML treatment significantly inhibited tumor growth in vivo, as shown by slower tumor growth rates in treated groups compared to controls, and regulated Pan Kla expression, correlating with decreased cancer cell proliferation	In vitro	[134]
Glycolysis	Fenbendazole (FZ)	Microtubules p53 Hexokinase II	Disruption of microtubule dynamics Increases p53 translocation to mitochondria, which is suggested to induce cell death Inhibition of HK2 activity, leading to apoptosis	FZ administration significantly reduced tumor size and weight in A549 xenografted nude mice	Animal studies	[135]
TCA cycle	Ivosidenib	Isocitrate dehydrogenase 1	Inhibits IDH1 catalysis of the oncometabolite 2HG that disrupts epigenetic regulation, blocks cellular differentiation, and contributes to tumorigenesis	Ivosidenib effectively suppresses plasma 2-HG in IDH1-mutated cholangiocarcinoma and chondrosarcoma, supporting a dose of 500 mg QD for advanced solid tumors	Phase I	[136]
Amino acid synthesis	JPH203 (Nanvuranlat)	L-type Amino Acid Transporter 1	LAT1 inhibition	The drug showed significant improvement in progression-free survival in patients with advanced, refractory biliary tract cancers compared to placebo, with a disease control rate of 25%; the treatment was found to be safe and well tolerated	Phase II	[137,138]
Glycolysis	Marinopyrrole derivative MP1	Myc and mTOR signaling	Modulate global gene expression and inhibit Myc-associated transcriptional targets including translation/mTOR targets. Inhibit tumor growth and Myc expression	MP1 is an orally bioavailable compound with favorable pharmacokinetics and pharmacodynamics, crossing the blood–brain barrier and achieving concentrations above IC50 in tumors, including in the brain, with good tolerability and no significant toxicity	Animal studies	[139,140]
Oxidative phosphorylation	Metformin	NADH	Increased flux of glucose carbons via the pentose phosphate pathway, leading to the inhibition of complex I (NADH:ubiquinone oxidoreductase)	Proneural BTICs respond better to metformin, while mesenchymal BTICs are more glycolytic and less responsive; glycolysis targeting may be more effective for mesenchymal BTICs.	Phase II	[141]
Glycolysis	Dimethylaminomicheliolide (DMAMCL), a Micheliolide derivative	Pyruvate kinase	Covalent binding at residue cysteine424 to promote tetramer formation and selectively activate PKM2	DMAMCL significantly suppresses tumor growth in vivo by activating PKM2, showing potential as a novel anticancer therapeutic drug, with optimal effects observed at 10 μg/mL	Discovery	[142]
Fatty acid synthesis	Omeprazole	Fatty acid synthase (FASN), which is a rate-limiting enzyme in synthesizing fatty acids	Proton pump inhibitors selectively inhibit FASN activity and induce apoptosis in Triple-Negative Breast Cancer (TNBC) cell lines via AKT and HIF-1 under hypoxic stress, allowing for adaptations in the tumor microenvironment	Omeprazole, when added to neoadjuvant AC-T, can safely inhibit FASN and shows a promising pCR rate, though further confirmation is needed	Phase II	[143]
Glycolysis	Oxamate	Lactate Dehydrogenase A	Induces inhibition of LDHA which suppress glucose uptake, lactate secretion, invasion, and proliferation in GH3 cells via the downregulation of GLUT1 and MMP2 expression and the inhibition of the Akt-GSK-3β-cyclinD1 pathway	Oxamate significantly inhibits the invasion and proliferation of primary pituitary PA cells derived from patients after transsphenoidal resection, confirming its potential as a therapeutic agent against human-invasive PA cells	Discovery	[144]
One-carbon metabolism	Methotrexate (MTX), Pemetrexed (PTX)	Serine hydroxymethyltransferases	Inhibit growth of cancer cells by cutting off the supply of 5,10-meTHF (utilized for nucleotide biosynthesis and hyperactivated in cancer)	PTX binds deeper in SHMTs than MTX due to its unique P-moiety structure, making it a more potent inhibitor; polyglutamylation significantly enhances the inhibitory activity of antifolates like PTX and MTX against SHMTs in vivo	Drug repurposing	[145]
Glycolysis	Shikonin	Pyruvate kinase	Decreases the PKM2-mediated aerobic glycolysis switch in tumor cells, thereby inhibiting tumor proliferation	Shikonin suppresses tumor growth in a dose-dependent manner in a mouse model of B16 melanoma at concentrations of 1 mg/kg and 10 mg/kg	Animal studies	[146]
Fatty acid synthesis	TVB 2640 (Denifanstat) + bevacizumab	Fatty acid synthase	Alternation of fatty acid synthase signaling which can drive phenotypic plasticity and cell fate decisions, mitochondrial regulation of cell death, immune escape, and organ-specific metastatic potential	TVB-2640 combined with bevacizumab significantly improved progression-free survival (PFS) in patients with recurrent high-grade astrocytoma compared to historical bevacizumab monotherapy, demonstrating a favorable safety profile and promising efficacy	Phase II	[147]
Amino acid synthesis	Venetoclax with azacitidine	BCL-2	Inhibition of BCL-2 which leads to the suppression of oxidative phosphorylation	Venetoclax and azacitidine show high response rates in treatment-naive patients, but relapsed patients exhibit reduced sensitivity due to metabolic adaptations in leukemic stem cells, indicating potential for targeting fatty acid metabolism	Phase I	[148]

Abbreviations: Hexokinase 2 (HK2); Oxaloacetic acid (OAA); reactive oxygen species (ROS); Monocarboxylate Transporter 1 (MCT1); Phosphoinositide 3-kinase (PI3K)/protein kinase B (AKT); Michigan Cancer Foundation-7 (MCF-7); MD Anderson-Metastatic Breast-231 (MDA-MB-231); Histone H3 lysine 9 lactylation (H3K9la); Histone H3 at lysine 56 lactylation (H3K56la); tumor protein 53 (p53); Isocitrate dehydrogenase 1 (IDH1); 2-hydroxyglutarate (2HG); L-type Amino Acid Transporter 1 (LAT1); Myelocytomatosis oncogene (Myc); mechanistic target of rapamycin (mTOR); Nicotinamide Adenine Dinucleotide (NADH); Pyruvate kinase M2 (PKM2); fatty acid synthase (FASN); Lactate Dehydrogenase A (LDHA); Hypoxia-Inducible Factor 1 Subunit Alpha (HIF1); growth hormone (GH3); glucose transporter type 1 (GLUT1); 5;10-methylenetetrahydrofolate (5;10-meTHF); Matrix Metallopeptidase 2 (MMP2); B-cell lymphoma 2 BCL-2 (BCL-2); Anthracycline–Taxane-based Chemotherapy (AC-T); Pathologic Complete Response (pCR); Pituitary Adenoma (PA); Serine Hydroxymethyl Transferase (SHMT); progression-free survival (PFS).
