# Correction: Chiu et al. Insights into Metabolic Reprogramming in Tumor Evolution and Therapy. *Cancers* 2024, *16*, 3513

**DOI:** 10.3390/cancers17040686

**Published:** 2025-02-18

**Authors:** Ching-Feng Chiu, Jonathan Jaime G. Guerrero, Ric Ryan H. Regalado, Jiayan Zhou, Kin Israel Notarte, Yu-Wei Lu, Paolo C. Encarnacion, Cidne Danielle D. Carles, Edrian M. Octavo, Dan Christopher I. Limbaroc, Charupong Saengboonmee, Shih-Yi Huang

**Affiliations:** 1 Graduate Institute of Metabolism and Obesity Sciences, Taipei Medical University, Taipei 110301, Taiwan; jgguerrero1@up.edu.ph (J.J.G.G.); yuway120@gmail.com (Y.-W.L.);; 2 Taipei Medical University Research Center of Cancer Translational Medicine, Taipei Medical University, Taipei 110301, Taiwan; 3 College of Medicine, University of the Philippines Manila, Manila 1000, Philippines; cdcarles@up.edu.ph (C.D.D.C.); emoctavo@up.edu.ph (E.M.O.); dilimbaroc1@up.edu.ph (D.C.I.L.); 4 College of Public Health, University of the Philippines Manila, Manila 1000, Philippines; 5 National Institute of Molecular Biology and Biotechnology, College of Science, University of the Philippines Diliman, Quezon City 1101, Philippines; rhregalado@up.edu.ph; 6 Department of Medicine, Stanford University School of Medicine, Stanford, CA 94305, USA; jyzhou@stanford.edu; 7 Department of Pathology, Johns Hopkins University School of Medicine, Baltimore, MD 21287, USA; kinotarte@gmail.com; 8 Department of Industrial Engineering and Management, Yuan Ze University, 135 Yuan-Tung Road, Chung-Li 32003, Taiwan; 9 Department of Biochemistry, Faculty of Medicine, Khon Kaen University, Khon Kaen 40002, Thailand; charusa@kku.ac.th; 10 School of Nutrition and Health Sciences, Taipei Medical University, Taipei 110301, Taiwan

## Authorship Change

Upon further reflection and in consultation with her academic advisors, Ms. Ma. Joy B. Zamora realized that her contribution to this publication was limited. Additionally, the main subject of the journal issue—cancer metabolism—does not align with her primary area of research as a master’s student who is focused on tardigrade proteins. As such, she believes it would be appropriate for her to be removed from the author list, as her involvement does not warrant authorship. The authors state that the scientific conclusions are unaffected. This correction was approved by the Academic Editor. The original publication [1] has also been updated.
